# Challenges with shifting, regardless of disengagement: attention mechanisms and eye movements in Williams syndrome

**DOI:** 10.1186/s11689-025-09639-z

**Published:** 2025-08-13

**Authors:** Astrid Hallman, Charlotte Willfors, Christine Fawcett, Matilda A. Frick, Ann Nordgren, Johan Lundin Kleberg

**Affiliations:** 1 Department of Psychology, Stockholm University, Frescativägen 8, Stockholm, 106 91 Sweden; 2 Department of Molecular Medicine and Surgery, Karolinska Institute, Stockholm, Sweden; 3 Department of Clinical Genetics and Genomics, Karolinska University Laboratory, Karolinska University Hospital, Stockholm, Sweden; 4 Department of Clinical Neuroscience, Karolinska Institute, Stockholm, Sweden; 5 Department of Psychology, Uppsala University, Uppsala, Sweden; 6 Child and Adolescent Psychiatry, Department of Medical Sciences, Uppsala University, Uppsala, Sweden; 7 Department of Laboratory Medicine, Institute of Biomedicine, University of Gothenburg, Gothenburg, Sweden; 8 Department of Clinical Genetics and Genomics, Sahlgrenska University Hospital, Gothenburg, Sweden

**Keywords:** Williams syndrome, Orienting attention, Pupil dilation, Eye tracking, Visual disengagement, Shifting attention, Phasic alerting effect, Intellectual disability

## Abstract

**Background:**

People with Williams syndrome (WS) face challenges in various areas of cognitive processing, including attention. Previous studies suggest that these challenges are particularly pronounced when disengagement of attention from a previously attended stimulus is required, as compared to shifting attention without the need to disengage. Difficulties with attention could in turn be implicated in several of the behavioral characteristics of WS. Here, disengagement and shifting of visual attention, together with pupil dilation, were independently assessed in one of the largest eye-tracking studies of WS to date.

**Methods:**

We investigated shifting, disengagement, and the effects of auditory alerting cues on pupil dilation in WS individuals (*n* = 45, age range = 9–58 years), non-WS individuals with intellectual disability (ID) (*n* = 36, age range = 6–59 years), and typically developed (TD) infants (*n* = 32, age range = 6–7 months), children and adults (*n* = 31, age range = 9–60 years), using a modified gap-overlap task. Data were analyzed using linear mixed-effect models (LMMs).

**Results:**

Individuals with WS were less likely to shift their attention to upcoming targets than TD individuals (all ages), but more likely than the ID group to do so. When they did shift attention, participants with WS and ID were slower to initiate a gaze shift than TD participants regardless of whether disengagement was needed. In the WS group, failure to shift attention was strongly predicted by higher arousal (pupil dilation), which was induced by auditory alerting cues.

**Conclusions:**

Contrasting with previous theories of attention in WS, we found no evidence for a specific challenge in disengaging attention. Instead, our results point to a more general challenge in shifting attention. Reduced attention shifting in WS may be partly explained by atypical arousal regulation. These results contribute to our understanding of the WS phenotype.

**Supplementary Information:**

The online version contains supplementary material available at 10.1186/s11689-025-09639-z.

Williams Syndrome (WS) is a rare genetic condition with a prevalence of around 1:7500 [1]. The condition arises from a micro-deletion on chromosome 7q11.23, and a majority of individuals with WS have an intellectual disability (ID) [2], mostly in the mild to moderate range [3]. The cognitive phenotype is characterized by relative strengths in verbal abilities, particularly concrete vocabulary, and verbal short-term memory, difficulties with spatial abilities [4], and challenges with executive function and attention [3, 5]. According to an influential hypothesis, WS is characterized by disproportionate challenges in disengagement of visual attention [6–11]. Individuals with WS exhibit prolonged attention to faces, resulting from difficulties disengaging attention from faces [12], but they do not focus on the eye region to the same extent as those without WS [13].

A strong attentional focus on faces and other social information may come at the cost of failing to attend to other important sources of information, such as the objects that other people attend to [14]. This selective challenge in disengagement has been likened to the pattern of *sticky fixation* observed in infants [9, 15]. Sticky fixation is hypothesized to explain a wide range of seemingly disparate phenomena within the WS phenotype, including relative expertise in face perception, unusual attention to others’ eyes, and challenges in visual perceptive skills, language learning, and numerosity. According to a complementary hypothesis, behaviors interpreted as reflecting disengagement difficulties are further exacerbated by physiological hypo-arousal [13, 16–18]. While these theories are not mutually exclusive, they make different predictions about the underlying mechanisms of the behavioral phenotype of WS. In the current study, we test these predictions using measures of disengagement, shifting, and phasic alerting in WS in one of the largest samples to date.

## How does attention disengagement impact visual processing and development?

Visual attention is currently understood as a set of interacting functions. An important distinction is between the orienting network, which directs attention toward prioritized sensory information (henceforth *orienting*), and the alerting network, which includes the arousal systems underlying attentional readiness, i.e., maintaining an aroused state to be prepared to attend to incoming stimuli or to sustain attention on a stimulus [19, 20]. Although the attentional networks typically interact, they are partly dissociable as evidenced by different developmental trajectories [21, 22] and neural correlates [19, 23]. Within the orienting network, a further distinction is made between disengagement from one stimulus, shifting attention toward the new input, and re-engaging focus on the new target, processes that involve distinct neural mechanisms [19]. Disengagement recruits the frontal eye fields (FEF) and intraparietal sulcus, shifting is mediated by the superior colliculus and temporoparietal junction, and re-engagement is mediated by thalamus [19]. While typically developing (TD) infants can overtly shift their attention from around three to four months of age, they still often show difficulty disengaging attention once fixated [21, 24]. The ability to disengage attention continues to improve throughout childhood and up into adolescence [25].

Since information taken in during visual fixations is heavily prioritized in all stages of visual information processing, eye movement atypicalities could potentially have widespread effects on behavior and cognition. For example, language learning could be affected by the inability to use joint attention to focus on relevant objects [26, 27], and self-regulation and emotional arousal could be affected by the inability to disengage from negative stimuli [28].

Building on this, and prior reports of disengagement difficulties in WS, it has been proposed that the syndrome’s unique profile of seemingly disparate strengths and challenges can partly be explained by a domain-general difficulty in planning and executing eye movements, which emerges early in life and continues throughout development [6, 9, 15].

## Is disengagement of attention atypical in Williams Syndrome?

The claim that disengagement difficulties are particularly prominent in WS has been made with reference to both social and non-social stimuli [6, 10, 12, 29]. Surprisingly, few studies presenting non-social stimuli used experimental designs that allowed for a distinction between disengagement and other aspects of visual attention. Most notably, without an appropriate experimental control condition, it is difficult to determine whether slower or reduced shifting of attention reflects challenges with disengagement alone or with shifting attention more broadly. For example, previous studies have shown that toddlers with WS were less likely to shift their gaze to a new target than those with Down syndrome [29], and children aged 3–41 months with WS produced fewer looks towards peripheral stimuli than children with Fragile X syndrome or age-matched TD children [6].

Disengagement difficulties in WS have also been explored in the context of social perception. For example, individuals with WS are slower to shift attention from centrally located social stimuli and show reduced attention to social stimuli in the periphery of a naturalistic scene compared to individuals with Fragile X syndrome, age-matched TD individuals, and TD individuals matched on mental age [10]. Additional findings supporting the disengagement difficulties hypothesis reported prolonged attention to socially salient information in WS compared to controls [8, 30–32]. While these findings suggest challenges with disengagement in WS, especially in response to social stimuli, alternative explanations must also be considered.

The idea that disengagement is the core problem of visual attention in WS was challenged in a recent study including infants and toddlers with WS, where the researchers did not find specific difficulties with disengagement of attention [26]. Compared to TD individuals or individuals with Down syndrome, individuals with WS were equally fast to shift to a peripheral non-social target regardless of whether they had to first disengage from a central stimulus. The authors suggested that this might be because infants and toddlers with WS do not fully focus on, or engage with, the central stimulus in the first place, reducing the need to disengage before shifting attention.

Several alternative explanations further complicate the interpretation of disengagement difficulties as a core attentional deficit in WS. For example, individuals with WS show longer reaction times when needing to disengage their attention from a location that previously showed a face rather than an object [12]. This could be a result of challenges with attention disengagement or reflect the increased reward value of social stimuli in WS [33]. The heightened social salience may lead to prolonged fixations that reflect increased subjective reward or relevance, rather than core difficulties in disengagement, complicating efforts to isolate disengagement as a distinct attentional difficulty in this population. As a comparison, in typical populations, saccadic eye movements are influenced by reward value and task relevance [34, 35], typically leading to quicker saccades to motivationally salient stimuli [35].

Another possible explanation comes from a predictive coding perspective. According to one proposal, covert attention may serve as a prediction that precedes and guides subsequent gaze shifts [36]. Prior knowledge or experience helps guide expectations, such as which objects usually appear in a natural scene, which words are likely to be most informative during reading, or where a target can appear [36]. Individuals with WS may struggle with forming or updating such predictions.

In sum, although multiple studies have reported findings consistent with disengagement difficulties in WS, this interpretation must be weighed against alternative explanations. Methodological differences and common use of tasks that confound disengagement with shifting further complicate the picture. Experimental designs detangling these factors and mechanisms might be beneficial for a more nuanced understanding of attentional processes in WS.

### Can hypo-arousal explain atypical attention in Williams Syndrome?

Building on the role of the alerting system in preparing the individual to respond to incoming stimuli, arousal and alertness emerge as critical components in shaping visual attention. Arousal can be understood as a global physiological and neural state that reflects overall levels of responsiveness to stimuli and influences alertness [37]. Alertness is defined as the cognitive state of readiness to respond to external stimuli [19], and alertness regulation has widespread effects on the speed and accuracy of visual attention [19, 38–41]. *Tonic alertness* refers to our general state of wakefulness or baseline arousal level over time. *Phasic alertness* refers to a rapid, temporary increase in alertness triggered by a warning cue, preparing the system to detect and respond to an upcoming stimulus. This quick rise in alertness can, in turn, decrease reaction times [19], enhance orienting to salient targets [42–44], and facilitate attention disengagement [39, 40]. The change in behavior (e.g., reaction time) in relation to the alerting cue is referred to as *the phasic alerting effect* and has already been documented in TD infants [45]. Phasic alerting is closely linked to the locus coeruleus-noradrenergic (LC-NE) system, which can be indirectly measured through pupil dilation [46, 47]. Pupil dilation reflects the intensity of the LC-activity, with a stronger alerting cue producing a larger pupil response and an increase in processing speed of visual information [38, 41]. The relationship between phasic alerting effects and LC-activity follows an inverse U-shaped curve: both low and high tonic alertness are linked to poorer performance, while intermediate levels optimize it [48]. Individuals with low tonic arousal often show stronger phasic responses, suggesting a compensatory mechanism [48].

Atypical arousal has been found in WS in several studies [13, 16–18, 49], where most consistently, these studies found evidence for hypo- rather than hyperarousal. So far, physiological arousal has mainly been examined in relation to social perception in WS, and little is known about how arousal interacts with visual attention beyond the social domain for this group. However, there is some evidence that the phasic alerting effect is stronger in children with neurodevelopmental conditions associated with atypical arousal regulation, such as children with ADHD [50]. This highlights the phasic alerting effect as a feasible marker for the effects of arousal on visual attention but has so far not been examined in WS.

## Conclusions and aims of the current study

Although there is clear evidence that individuals with WS have challenges with visual attention, support for the hypothesis that shifting with disengagement as compared to shifting more generally underlies these challenges is less robust. The majority of studies reporting disengagement challenges in WS have used social stimuli and, in many cases, tasks that do not allow differentiation between disengagement and shifting. Although both reduced tonic and phasic arousal have been suggested to underlie altered attention in WS, there is little evidence for this claim in areas other than social perception. Furthermore, the majority of studies correlated attention-relevant behaviors with measures of tonic arousal but did not manipulate phasic arousal. Firm conclusions about the nature of visual attention alterations in WS are also limited by the fact that most studies examined small groups of participants, ranging from 8 to 25 participants [6, 7, 12, 26, 27].

The current study independently assessed disengagement, shifting, and phasic alerting using a validated measure previously used in studies of other neurodevelopmental conditions: the gap-overlap task. We also aimed to examine atypical aspects of the developmental trajectory for the WS phenotype. For this purpose, we compared the performance of participants with WS to that of TD individuals covering the age span from infancy to adulthood, as well as to that of an ID group with other genetic syndromes than WS. The 6- to 7-month-old infant group specifically was important to include because infants at this age are beginning to disengage their attention more easily than younger infants and start to show the phasic alerting effect [45]. Although the adults’ shifts are faster, similar reflexive orienting attention patterns have been observed between 6-month-old infants and adults [51]. This comparison allowed us to explore whether attentional profiles in WS reflect developmental delay (i.e., resembling an earlier typical developmental stage) or atypical development (i.e., patterns not observed at any typical developmental stage).

Disengagement efficiency is the relative decrease in efficient shifting when disengagement is also necessary and was assessed by comparing gaze shift latency between the gap condition (i.e., shifting only) and overlap condition (i.e., shifting with disengagement) [25, 52]. In neurotypical development, disengagement efficiency increases with age until adolescence [25] and is minimally affected by age afterwards [53]. The measure has good psychometric properties in infants [52] and adolescents [25].

We hypothesized that individuals with WS would face specific challenges in shifting their gaze when disengagement is necessary (i.e., during overlap trials specifically) compared to both TD individuals and individuals with ID of a genetic origin other than WS. This hypothesis is based on previous suggestions that disengagement difficulties may contribute to the attentional atypicalities seen in WS. Alternatively, if reduced *shifting* of visual attention broadly characterizes WS, regardless of disengagement demands, we would expect to observe increased gaze shift latencies in both gap and overlap trials. Finally, we hypothesized that enhancing arousal through a phasic alerting cue would facilitate disengagement, leading to reduced gaze shift latencies. If reduced arousal contributes to disengagement challenges in WS, we reasoned that such a manipulation might partially regulate this behavior.

## Methods

### Participants

The study was part of a larger ongoing research project examining behavioral phenotypes of rare genetic conditions. Participants with WS were tested between 2018 and 2023, and participants in the non-WS ID group were tested between 2018 and 2024. The total sample comprised a maximum of 144 participants, although the sample size was slightly smaller for some of the analyses (see Table 1). Data was collected in lab settings and at patient organizations’ family gatherings.

*WS*: Initially, 49 individuals with WS aged 8 to 57 years old participated. Advertisements were made through patient and family organizations and health care services. Four individuals were excluded due to not providing any valid eye-tracking data. This left a final sample size of 45 individuals with WS aged 9 to 57 years old, although the sample size was slightly smaller for some of the main analyses, see Table 1 for further information. Twenty-two individuals and their caregivers participated in a larger assessment, which included a diagnostic interview regarding psychiatric conditions and neuropsychological testing conducted by a clinical psychologist or child and adolescent psychiatrist [54]. Except for ID, co-occurring psychiatric conditions were autism (*n* = 3), ADHD (*n* = 4), tics disorder (*n* = 1), depression (*n* = 2), panic disorder (*n* = 4), obsessive-compulsive disorder (OCD) (*n* = 2), post-traumatic stress disorder (PTSD) (*n* = 1), generalized anxiety disorder (GAD) (*n* = 3), specific phobia (*n* = 11), and anxiety disorder not otherwise specified (*n* = 3). Information regarding ID severity was available for 21 individuals: mild (*n* = 15) and moderate (*n* = 6).

*ID*: Forty-nine individuals with a rare genetic condition other than WS and co-occurring ID aged 6 to 59 years old were recruited. From here, the group is referred to as the ID group. Twelve individuals were excluded due to not providing any valid eye-tracking data (Coffin-Siris *n* = 1, FXS *n* = 4, Smith-Magenis syndrome *n* = 2, DS *n* = 5). One individual was removed due to not having an ID. The final sample consisted of 36 individuals (22q11 deletion syndrome *n* = 3, Coffin-Siris syndrome *n* = 6, DS *n* = 7, FXS *n* = 7, Smith-Magenis syndrome *n* = 9, Sotos syndrome *n* = 3, Other rare deletion *n* = 1). Information regarding ID severity was available for 15 individuals: mild (*n* = 6), moderate (*n* = 8), and unspecified (*n* = 1). Co-occurring psychiatric and neurologic conditions were autism (*n* = 4), ADHD (*n* = 8), epilepsy (*n* = 1), language disorder (*n* = 3), OCD (*n* = 1), GAD (*n* = 1), and agenesis of the corpus callosum (*n* = 1). Information regarding ID severity and co-occurring conditions was acquired through parental reports. Advertisement was done through patient and family organizations and health care services.

### TD children and adults

Initially, 40 individuals with no somatic, neurologic, or psychiatric condition, further referred to as TD, were recruited via an advertisement in social media and a database for people who have expressed interest in participating in research. Information regarding medical and psychiatric conditions was acquired through parental reports or self-reports. Participants older than 60 years of age (*n* = 3) were excluded from the analysis to match the age range of the WS and ID participants. Six individuals were removed from the main analysis due to an administration error. This leaves a final sample of 31 TD participants aged 9 to 60 years.

### TD infants

Thirty-nine TD infants (age range 6 to 7 months) were recruited through a database of families who have expressed interest in participating in research. Seven participants were excluded from the analysis due to one parent having a neurodevelopmental condition (*n* = 4), gestational age less than week 35 (*n* = 1), birth complications (*n* = 1), and suspected genetic syndromes in multiple second-degree relatives (*n* = 1). The final sample consisted of 32 participants.

## Materials and procedure

For identifying adaptive functioning in everyday life in the syndrome groups, the standard score of Global Adaptive Functioning (GAF) from the Swedish version of Adaptive Behavior Assessment Scales [55] was used.

### Procedure and experimental paradigm

The gap-overlap task is a commonly used experimental paradigm used to disentangle the subcomponents of orienting of attention [26, 56–58]. For this study, the task has been modified to include auditory cues with the aim of briefly increasing arousal. Throughout the task, the auditory cues were not associated with particular visual cues or locations [40]. In order to be able to investigate the phasic alerting cues’ effect on disengagement, the auditory cues were only used on the trials where disengagement was needed, the overlap trials.

**Fig. 1:**
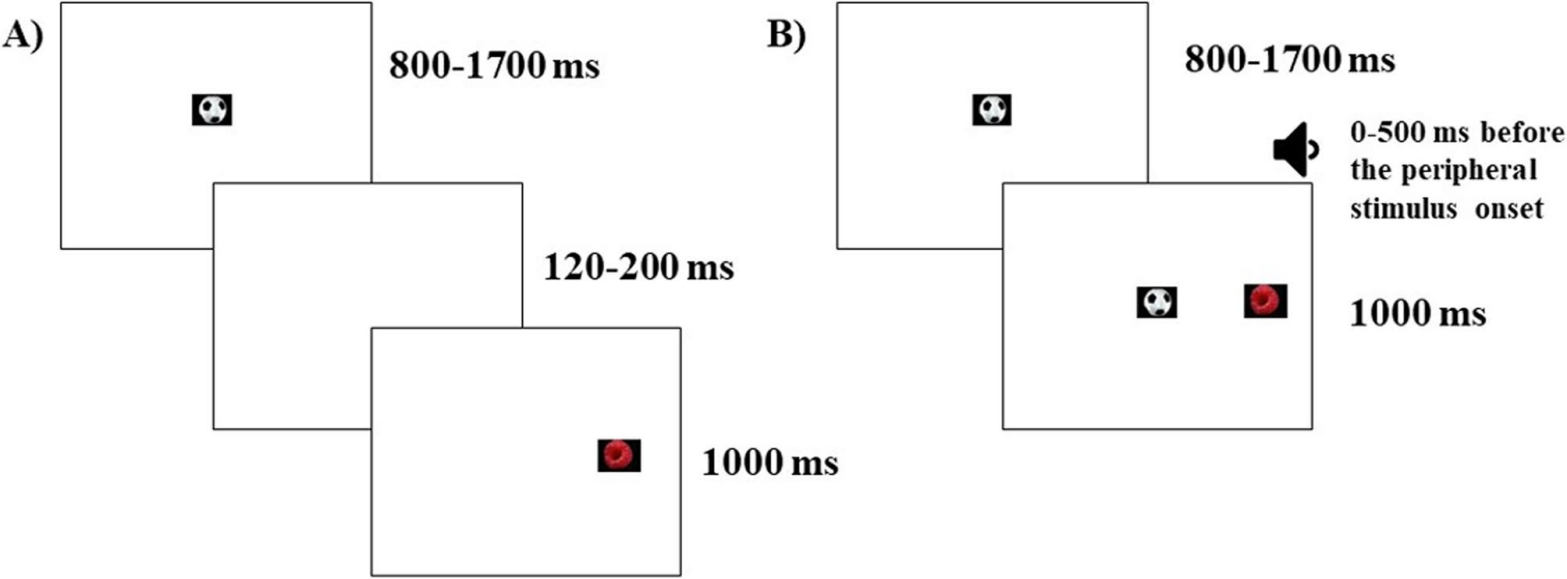
Illustration of a gap and an overlap trial. **A** The gap trials. The central stimulus is present on the screen for an interval ranging between 800 and 1700 milliseconds (ms). The central stimulus disappears for 120-200 ms, creating a temporal gap. The peripheral stimulus appears for 1000 ms. **B** The overlap trials. The central stimulus is present for the same time range as on the gap trials. The peripheral stimulus then appears, together with the central stimulus, for 1000 ms. On the overlap cued trials, the onset of the auditory alerting cue varies between 0-500 ms before the onset of the peripheral stimulus.

The resulting effects on pupil dilation and gaze behavior give an index of phasic alerting. The task consisted of three conditions: gap, overlap silent, and overlap cued trials. In the gap trials, the central target disappeared before the onset of the peripheral target, creating a temporal “gap”. In the overlap trials, the central stimulus remained on the screen during the onset of the next target, creating a temporal “overlap”. In the cued overlap trials, the trials were combined with auditory stimuli. See Fig. 1 for an illustration of a trial for each condition. The auditory stimuli consisted of spoken interjections such as “oh!” and beeps. The visual stimuli consisted of images of non-social everyday objects, such as a football or a raspberry. All visual stimuli subtly blinked, flickered, or moved to capture the participants’ attention. The participants received the following instructions, in Swedish, at the beginning of the task: *Look at the pictures appearing on the screen.* For this task, 58 trials were presented in total (gap: 21, overlap silent: 18, overlap cued: 19; the slightly different number of trials per condition resulted from a programming error). Three areas of interest (AOI) were identified. One AOI covered the fixation cross displayed in the middle of the screen, while two AOIs covered the peripheral targets, one on the left side and one on the right side.

### Recording and processing of eye-tracking data

Eye movements and pupil data were recorded with two different eye trackers; a Tobii Pro Spectrum with a sampling rate of 1200 Hz (*n* = 35) and a Tobii X120 with a sampling rate of 120 Hz (*n* = 108) (Tobii Technology AB, Danderyd, Sweden). Pupil data were recorded at 40 HZ (Tobii X120) and 1200 HZ (Tobii Pro Spectrum). To identify fixations, an I-VT filter with a velocity threshold set to 30°/s and window length set to 20 ms was used.

Pupil data were processed following the procedure by Kleberg et al. [40]. Linear interpolation was used to cover gaps shorter than 100 ms. A moving median filter was used for filtering, with a window size of 80 ms. Pupil dilation amplitude was calculated as the median pupil size during a 0–1500 ms time window following the onset of the peripheral stimuli (the response period). This measurement was baseline-corrected by subtracting the median pupil size observed during a baseline period of 333 ms. Following recent recommendations [59, 60], baseline correction was conducted through subtraction. The baseline period was defined as the 1000–666 ms interval before stimulus onset, i.e., before the onset of both the alerting cues and the peripheral visual stimuli. Consistent with [40], we selected a uniform baseline interval for all trials rather than a specific period time-locked to each sound to facilitate comparison between the cued and uncued conditions. Trials with less than 30% valid samples were discarded. For detecting outliers on the pupil response variable, 3* Median Absolute Deviation (MAD) was used per participant [61]. 10.6% of trials from the WS group, 13.1% of the ID group, 6.9% of the TD group and 12.9% of the infant group were identified and removed.

Saccades that occurred with a latency of less than 100 ms were deemed anticipatory and, thus, excluded from the analysis. Saccades with a latency above 1000 ms were interpreted as improbable values and excluded from the analysis. This threshold was set just above the 99th percentile (933 ms), ensuring that only the most extreme 1% of responses were removed, while retaining the vast majority of the data. In order to keep as many valid trials as possible for each analysis, participants with fewer than four valid trials in any experimental condition were removed from the analyses [WS: gap: *n* = 1; overlap silent: *n* = 1 overlap cued: *n* = 2; ID: gap: *n* = 4; overlap silent: *n* = 2; overlap cued: *n* = 4; TD: no participants were removed; TD infant: no participants were removed]. See Table 1 for the number of valid trials per outcome measure.

### Dependent variables

Gaze shift latency was measured as the response time in ms for the gaze to shift towards the peripheral AOI from the central AOI. Disengagement efficacy (gap effect) was assessed by estimating model-based contrasts between the gap and overlap conditions using the emmeans package [62]. Similarly, the cue effect (phasic alerting effect) was examined by estimating the contrast in saccadic reaction time between the overlap silent and overlap cued conditions. These contrasts allowed us to evaluate differences in gaze shift latency across conditions while accounting for fixed and random effects. Percentage of no-shift trials refers to the percentage units of trials where no saccade from the central AOI occurred, providing further insight into disengagement cost. Pupil dilation was defined as the change in pupil diameter (mm) from baseline in response to the alerting cue during the overlap conditions.

### Statistical analysis

Since participants had varying valid trials per condition, data was analyzed with linear mixed-effects models (LMMs), on trial level with participant as varying effect and group (WS, ID, TD, and TD-infant) and condition (the three experimental conditions) as fixed effects^1^. LMMs were performed in R [63] (version 4.2.0) using the lme4 package [64].

Follow-up comparisons were made with the emmeans package [62] in R on both condition and group, as well as between conditions within each group. Bonferroni corrected *p*-values are reported for all follow-up tests, and the degrees of freedom were adjusted using the Satterthwaite method. The level of statistical significance was set at 0.05. Gaze shift latencies were log-transformed for the analysis and are presented in log(ms). Visualizations were conducted using the ggplot2 package [65].

### Pre-registered analysis plan

An analysis plan was preregistered at Open Science Framework (OSF https://osf.io/qkam3/?view_only=f8b9257e73824969af67e7c6ca6b65ac) during data collection and before examination of the data. An Analysis of Variance (ANOVA) was originally intended as the primary statistical analysis method. However, given the variability in valid trials across experimental conditions, linear mixed-effect models (LMMs) were used. A comparison group of TD infants was added to the analysis to investigate if the attention profiles in WS differ early in the development of attention.

## Results

### Preliminary analysis

Demographic data and the mean GAF score from the WS (*n* = 19) and ID group (*n* = 17) are presented in Table 1. Normality was inspected using visualization and tested using the Shapiro-Wilks test. Since normality was not met for age (WS: *W* = 0.89, *p* <.001; ID: *W* = 0.92, *p* <.001; TD: *W* = 0.92, *p* <.001; TD infant: *W* = 0.92, *p* <.001) Kruskal-Wallis test was used for group comparisons. Follow-up pairwise comparisons were conducted using Wilcoxon rank-sum tests. Fisher’s exact test was used for pairwise group comparisons between binary variables. As can be seen in Table 1, the proportion of female participants was higher in the TD group than in the WS (*p* =.009), ID (*p* <.001), and TD infant (*p* =.005) groups. There was a difference in age across groups (χ^2^ (3) = 3364, *p* <.001). The TD group had generally older participants (all: *p* <.001), and the participants in the WS group were older than the participants in the ID group (*p* <.001). For results regarding mean pupil baseline, see Supplementary material.

**Table 1:** Sample characteristics

Group	WS	ID	TD	TD infants	Group differences
	M (SD)	M (SD)	M (SD)	M (SD)	
Age in years	25.0 (12.4)	19.7 (10.5)	28.4 (16.6)	0.58 (0.02)	ID < WS < TD
Sex, F/M, *n/n*	24/21	16/20	22/9	18/14	TD > ID|WS|TD infant
*N*	45	36	31	32	
GAF	66 (10.3)	60 (8.6)	-	-	WS > ID
Pupil baseline (mm)	3.24 (0.55)	3.31 (0.47)	3.04 (0.32)	3.32 (0.41)	TD < WS < ID|TD-infant
Valid trials					
*Gaze Shift Latency*					
* N* included	43	26	31	32	
Gap	15.4 (3.77)	13.6 (3.61)	18 (1.68)	12.2 (3.06)	
Overlap S	14.1 (3.55)	11.9 (3.61)	17 (1.38)	11.5 (3.00)	
Overlap C	13.8 (3.37)	11.8 (3.28)	16.8 (0.91)	11.4 (2.97)	
*Percentage No shift trials*					
* N* included	44	33	31	32	
Gap	17.2 (2.60)	14.4 (4.05)	18.3 (1.57)	13.8 (2.69)	
Overlap S	16.5 (2.70)	13.7 (3.62)	17.5 (1.36)	14.5 (2.41)	
Overlap C	17.4 (2.45)	15.2 (3.61)	18.6 (1.33)	15.8 (2.23)	
*Pupil dilation*					
* N* included	43	26	31	32	
Gap	16.3 (3.14)	13.9 (4.26)	17.8 (1.87)	11.8 (2.97)	
Overlap S	15.7 (3.00)	12.9 (3.55)	17.2 (1.85)	12.8 (3.11)	
Overlap C	16.7 (2.45)	14.1 (3.60)	17.8 (2.00)	13 (2.83)	
*Sound type*					
Simple	8.72 (2.03)	7.19 (2.48)	9.74 (1.26)	8 (1.76)	
Complex	7.91 (1.82)	6.55 (2.38)	8.71 (0.69)	7.44 (1.24)	

Standard deviations are in parentheses. *WS* Williams syndrome, *TD* Typically developed, *ID* Intellectual disability, *GAF* Global Adaptive Functioning, *Overlap S* Overlap silent, *Overlap C* Overlap cued. The TD infant group is not included in age comparisons since they are outside the other groups’ age range. Valid trials are presented as the mean number of valid trials per participant for each outcome measure. Sound type refers to the mean number of valid trials per sound type in the overlap cued condition

Bivariate linear regression models were used to analyze the effect of sex and age on the outcome measures within each group. Results showed that age and sex had no significant impact on gaze shift latency (age: all *p* >.422; sex: all *p* >.621), percentage of no-shift trials (age: all *p* >.172; sex: all *p* >.255), or pupil response (age: all *p* >.273; sex: WS, ID and TD *p* >.105), except for TD infant where sex had an impact on pupil response (*b* = 0.03, SE = 0.01, *p* =.025). Since neither of the three other groups had an effect of sex, age and sex were not included as covariates in the models.

### Gaze shift latency

There was a significant main effect of condition (*F*(2, 4820.7) = 136.45, *p* <.001) on gaze shift latency, indicating that gaze shift latency differed significantly across the three conditions. Pairwise follow-up comparisons showed that the overlap silent condition showed an increase in gaze shift latency (*b* = −0.17, SE = 0.01, *p* <.001) as did the cued overlap condition (*b* = −0.22, SE = 0.01, *p* <.001), both compared to the gap condition. There was also a significant difference in gaze shift latencies between the cued and silent overlap conditions (*b* = −0.05, SE = 0.01, *p* =.002), with participants showing slower responses on the overlap cued condition compared to the silent one. There was a significant main effect of group (*F*(3, 121.4) = 12.82, *p* <.001) on gaze shift latency, suggesting that the four groups differed in their gaze shift latencies. Pairwise follow-up comparisons showed that the TD and TD infant groups exhibited significantly faster gaze shift latencies than the WS group (TD: *b* = 0.21, SE = 0.05, *p* <.001; TD infant: *b* = 0.27, SE = 0.05, *p* <.001) and the ID group (TD: *b* = 0.23, SE = 0.06, *p* =.002; TD infant: *b* = 0.29, SE = 0.06, *p* <.001). The ID group showed no significant difference compared to the WS group (*b* = −0.02, SE = 0.06, *p* >.999). The TD group showed no significant difference compared to the TD infant group (*b* = 0.06, SE = 0.06, *p* >.999). There was a significant interaction effect for condition and group (*F*(6, 4817) = 2.21, *p* =.039), indicating that the effect of condition on gaze shift latencies varied across the different groups.

**Fig. 2:**
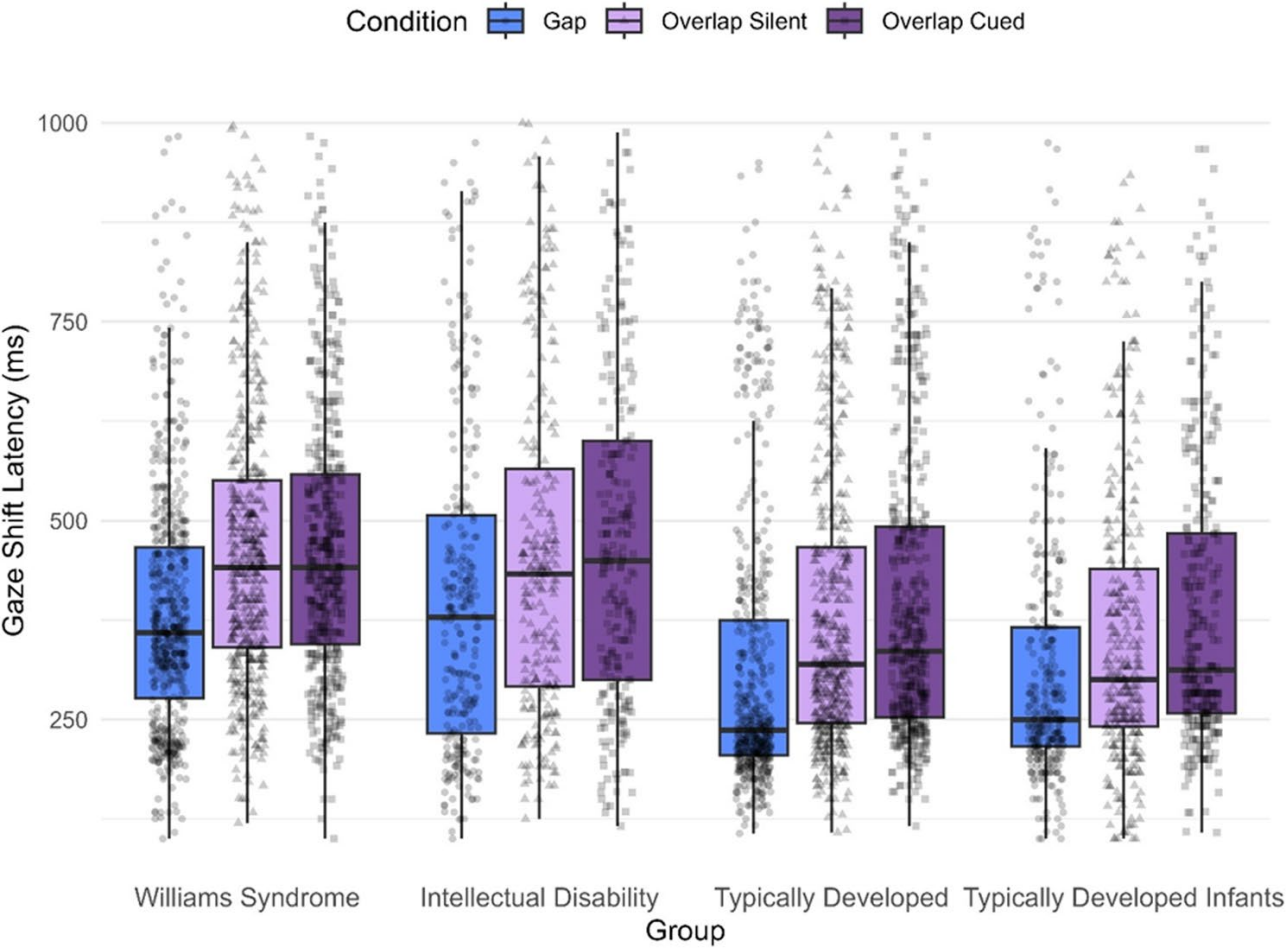
Gaze shift latency (ms) by group and condition. Mean gaze shift latency on gap, overlap silent, and overlap cued trials for individuals with Williams syndrome, individuals with intellectual disability of mixed genetic origin, typically developed individuals, and typically developed infants across conditions for each group. Gaze shift latency is measured in milliseconds (ms) from when the participants shift their gaze from the central AOI to the peripheral AOI. Data points are shown in raw values

To examine within-group effects, we performed post-hoc pairwise comparisons across conditions. This allowed us to assess disengagement efficiency and to determine whether a phasic alerting effect, indicated by shorter gaze shift latencies in the cued relative to the silent overlap condition, was evident within each group. Within-group comparisons revealed that, as shown in Fig. 2, all groups exhibited significantly slower gaze shift latencies in both overlap conditions compared to the gap condition (all *p* <.001). However, the TD infant group was the only group that exhibited significantly faster gaze shift latencies in the overlap silent condition compared to overlap cued (TD-infants: *b* = 0.88, SE = 0.03, *p* =.006; other groups: *p* >.185).

Comparisons of contrasts examining within-condition differences across groups showed no significant differences (all *p* >.702), suggesting that the magnitude of the condition effects (e.g., gap vs. overlap silent) did not vary among any of the groups (see Supplementary Materials, Table S9).

As can be seen in Fig. 2; Table 2, comparisons between groups within each experimental condition, revealed that the WS and ID groups did not differ in gaze shift latencies in any of the experimental conditions (all *p* >. 999), and neither did the TD and the TD infant groups (all *p* >.508). The TD group had significantly faster gaze shift latencies than both the WS group (all *p* <.013) and the ID group (all *p* <.024), as did the TD infant group (WS: all *p* <.001; ID: all *p* <.001) in all conditions.

Together, this indicates that if a syndrome with co-occurring ID is present, the gaze shift latencies will be significantly slower than if a syndrome is not present.

**Table 2:** Pairwise comparisons of gaze shift latency in the ratio of Ms across group and condition

Group comparisons	Ratio of ms	SE	df	t.ratio	*p*-value
*Gap*					
WS - ID	0.95	0.06	174	−0.76	> 0.999
WS - TD	1.27	0.07	153	4.23	**< 0.001**
WS - TD infant	**1.29**	0.07	172	4.31	**< 0.001**
ID - TD	**1.33**	0.08	164	4.40	**< 0.001**
ID – TD infant	**1.35**	0.09	180	4.47	**< 0.001**
TD - TD infant	1.01	0.06	162	0.16	> 0.999
*Overlap Silent*					
WS - ID	1.02	0.06	180	0.29	> 0.999
WS - TD	**1.24**	0.07	158	3.70	**0.002**
WS - TD infant	**1.38**	0.08	175	5.48	**< 0.001**
ID - TD	**1.22**	0.08	169	2.95	**0.022**
ID – TD infant	**1.35**	0.09	183	4.52	**< 0.001**
TD - TD infant	1.11	0.07	164	1.74	0.508
*Overlap Cued*					
WS - ID	0.99	0.06	186	−0.23	> 0.999
WS - TD	**1.20**	0.07	160	3.12	**0.013**
WS - TD infant	**1.27**	0.07	176	4.09	**< 0.001**
ID – TD	**1.21**	0.08	172	2.92	**0.024**
ID – TD infant	**1.29**	0.08	185	3.78	**0.001**
TD - TD infant	1.06	0.07	163	0.96	> 0.999

*SE* Standard error, *TD* Typically developed, *WS* Williams syndrome, *ID* Intellectual disability. Latency is presented in the difference in the ratio of ms comparing the estimated means between conditions. A ratio value less than 1 indicates that the first condition has a lower estimated mean than the second condition, and a ratio greater than 1 indicates the opposite. Bold indicates a *p*-value below 0.05

### Percentage of no-shift trials

Results are presented in mean percentage. There was a main effect of condition (*F*(2, 6224.5) = 53.76, *p* <.001), indicating that there was a difference in the percentage of no-shift trials between the conditions. Pairwise follow-up comparisons showed that the participants had a higher percentage of no-shift trials in the overlap conditions (overlap silent: *b* = −5.04, SE = 1.14, *p* <.001; overlap cued: *b* = −11.56, SE = 1.12, *p* <.001) compared to the gap condition but also in relation to each other with a higher percentage of no-shift trials in the cued overlap condition compared to the silent (*b* = −6.52, SE = 1.12, *p* <.001). There was a main effect of group (*F*(3, 129.5) = 13.81, *p* <.001), indicating that there was a difference between the groups regarding mean percentage of no-shift trials. See Table 3 for estimated mean values per group and condition.

**Fig. 3:**
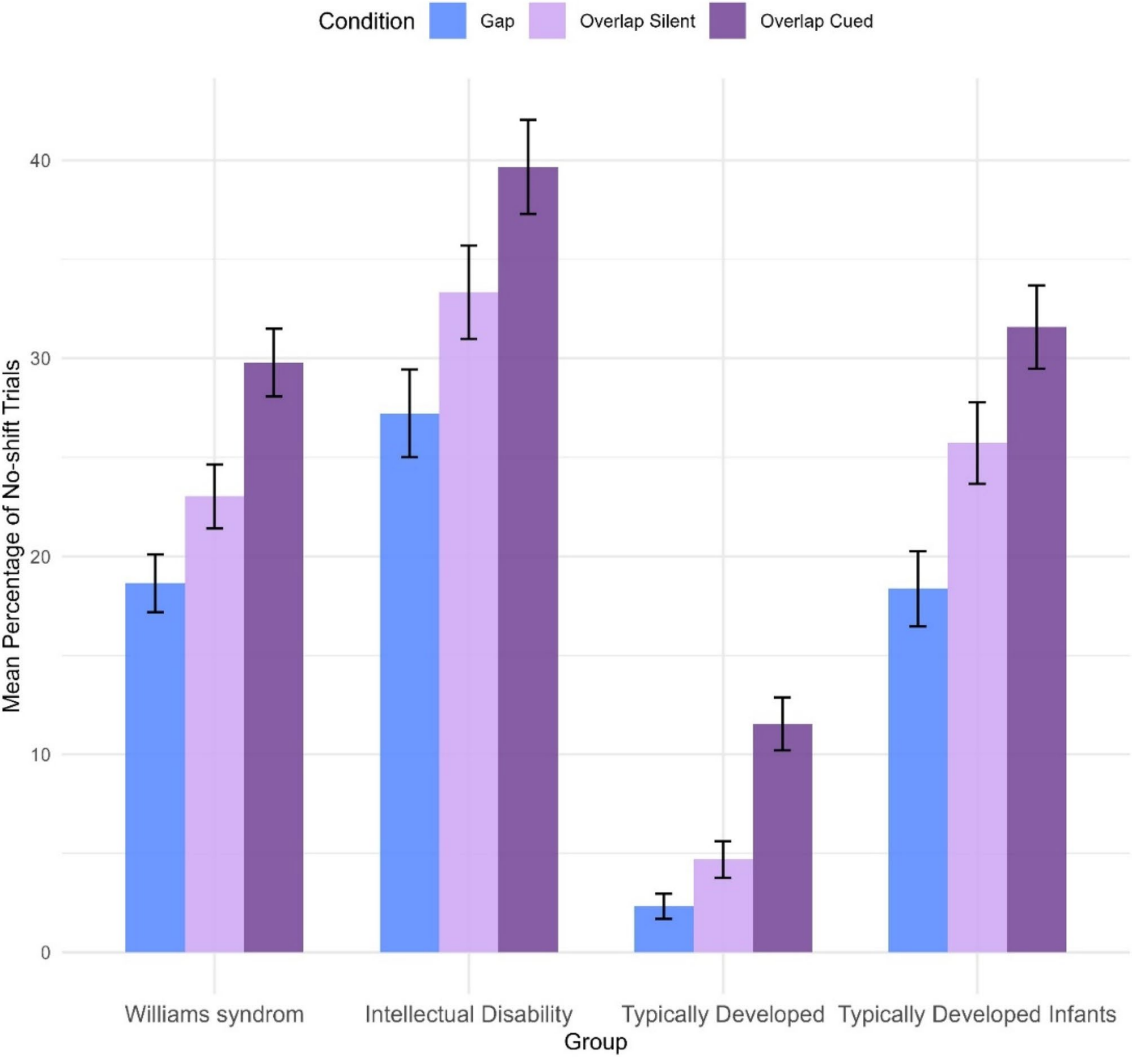
Percentage of no-shift trials across group and condition. Mean percentage units of no-shift trials on gap, overlap silent, and overlap cued trials for individuals with Williams syndrome, individuals with intellectual disability of mixed genetic origin, typically developed individuals and typically developed infants across conditions for each group. Error bars represent ±1 standard error of the mean (SEM)

Pairwise follow-up comparisons (Fig. 3) showed that the WS group had a higher percentage of no-shift trials than the TD group (*b* = 18.63, SE = 4.62, *p* <.001) but not the TD infant group (*b* = −0.67, SE = 4.60, *p* >.999) and a significantly lower percentage of no-shift trials than the ID group (*b* = −13.07, SE = 4.61, *p* =.031). In comparison, the ID group had a significantly higher percentage of no-shift trials than the TD group (*b* = 31.70, SE = 4.97, *p* <.001) but not the TD infant group (*b* = 12.40, SE = 4.95, *p* =.081). The TD group had a significantly lower percentage of no-shift trials than the TD-infant group (*b* = −19.30, SE = 4.97, *p* =.001). There was no group-by-condition interaction effect (*F*(6, 6223.6) = 0.52, *p* =.793). For explorative pairwise comparisons, see Table S8 in Supplementary Materials.

**Table 3:** Estimated mean percentage of no-shift trials per group and condition

Condition	Mean	SE	df
*WS*			
Gap	20.14	3.18	173
Overlap Silent	25.07	3.19	175
Overlap Cued	31.69	3.18	173
*ID*			
Gap	32.67	3.80	197
Overlap Silent	38.11	3.79	196
Overlap Cued	45.33	3.79	193
*TD*			
Gap	3.22	3.74	165
Overlap Silent	5.59	3.75	167
Overlap Cued	12.22	3.73	163
*TD-infant*			
Gap	19.48	3.79	186
Overlap Silent	26.92	3.76	180
Overlap Cued	32.51	3.73	174

*SE* Standard error, *TD* Typically developed, *WS* Williams syndrome, *ID* Intellectual disability. Percentage no-shift trials are presented in percentage units of trials where the participant does not shift their gaze to the periphery

### Pupil dilation

When analyzing the effect of an auditory alerting cue on the overlap conditions, the analysis was only conducted on the two overlap conditions, which are identical in terms of visual information (see Methods), thus controlling for potential confounding effects on pupil size. There was a main effect of condition (*F*(1, 3400.5) = 104.51, *p* <.001), driven by larger pupil responses on cued than silent trials. There was no significant main effect of group (*F*(3, 120.2) = 1.19, *p* =.315), indicating no overall significant difference in pupil response between the groups. However, there was a group-by-condition interaction effect (*F*(3, 3393.1) = 4.26, *p* =.005).

To assess within-group effects, we conducted post-hoc pairwise comparisons between conditions for each group to determine if a pupil response was present within that group. Within-group comparisons indicated that all groups displayed a greater pupil response on the overlap cued condition compared to the silent overlap trials, with the exception of the ID group (*b* = −0.02, SE = 0.01, *p* =.152; all other groups *p* <.001).

To further examine whether the magnitude of the pupil response differed between groups, we compared within-group contrasts across groups. These contrast of contrasts comparisons showed that the ID group exhibited a significantly smaller difference in pupil dilation between the overlap conditions compared to the WS (Estimate = −0.044, SE = 0.02, *p* =.025) and TD infant (Estimate = 0.057, SE = 0.02, *p* =.003) groups, and a marginally smaller difference compared to the TD group (Estimate = 0.041, SE = 0.02, *p* =.056). No other between-group differences in contrast magnitude were significant (all *p* >.999). These results indicate that the ID group’s blunted pupil response to auditory cues presence likely underlies the observed contrast differences with other groups. (See Supplementary Materials, Table S10).

**Fig. 4:**
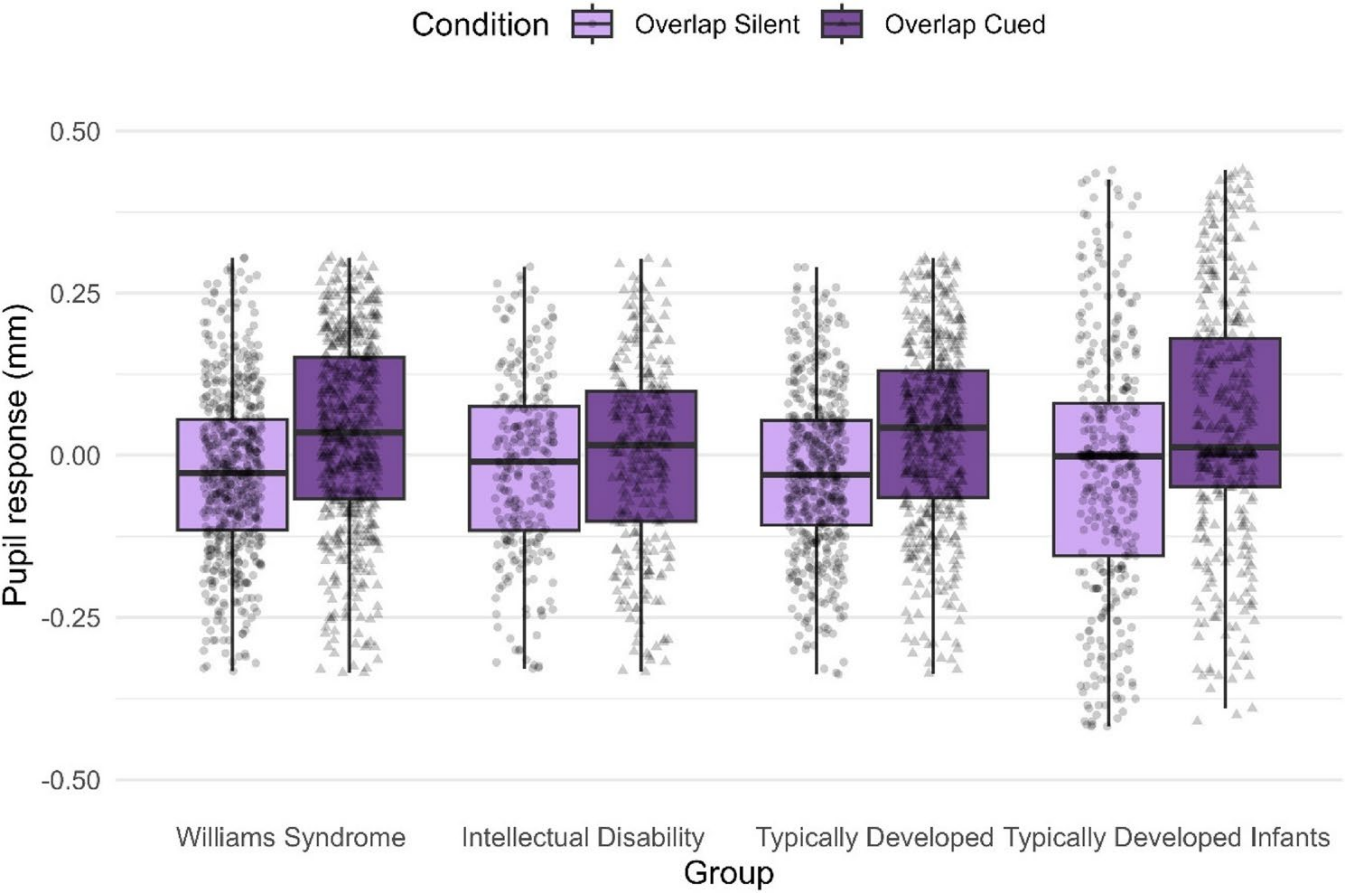
Pupil dilation across group and condition. Mean pupil dilation across the conditions overlap silent and overlap cued for each group. Pupil dilation is measured in change in millimeters (mm) in relation to baseline

As can be seen in Fig. 4, pairwise post hoc group comparisons for the overlap cued condition revealed a significant difference in pupil response between the WS and the ID group (*b* = 0.04, SE = 0.01, *p* =.040) and between the ID and the TD infant group (*b* = −0.05, SE = 0.01, *p* =.002). This indicates that individuals in the ID group, on average, showed a significantly smaller pupil response to the alerting cue than individuals in the TD infant and WS groups. No other between-group differences were significant (all *p* >.079). No group differences emerged on the silent overlap condition (all *p* >.999, see Table 4), supporting the notion that these group differences were elicited by the alerting cue.

**Table 4:** Pairwise comparisons of pupil dilation in mm across group and condition

Group comparisons	Difference estimate	SE	df	t.ratio	*p*-value
*Overlap Silent*					
WS - ID	−0.008	0.013	312	−0.610	> 0.999
WS - TD	−0.001	0.011	225	−0.111	> 0.999
WS – TD infant	−0.001	0.012	285	−0.114	> 0.999
ID - TD	0.007	0.014	284	0.498	> 0.999
ID – TD infant	0.007	0.014	334	0.471	> 0.999
TD - TD infant	< 0.001	0.013	258	−0.009	> 0.999
*Overlap Cued*					
WS - ID	**0.035**	0.013	284	2.734	**0.040**
WS - TD	0.002	0.012	230	0.149	> 0.999
WS – TD infant	−0.015	0.012	278	−1.215	> 0.999
ID - TD	−0.034	0.014	266	−2.496	0.079
ID – TD infant	**−0.050**	0.014	306	−3.588	**0.002**
TD – TD infant	−0.016	0.013	259	−1.294	> 0.999

*SE* Standard error, *TD* Typically developed, *WS* Williams syndrome, *ID* Intellectual disability. Pupil dilation is presented in difference in mm in diameter in relation to the baseline. Bold indicates a p-value below 0.05

### Does pupil dilation predict the likelihood of shifting attention?

An exploratory analysis was conducted to investigate the effect of pupil response on the likelihood of not shifting attention in the overlap cued condition. Separate generalized linear mixed models (GLMM) with participant as a random effect and pupillary response and percentage of no-shift trials on the overlap cued condition as fixed effects were performed for each group. Bonferroni correction for multiple comparisons was applied to the p-values.

The results show varying effects of pupil response across the different groups. Individuals with WS and TD^2^ show a substantial effect (WS: OR = 16.87, SE = 0.75, *p* =.001; TD: OR = 39.28, SE = 1.12, *p* =.004), indicating that the odds of not shifting attention in the overlap cued condition increased with a higher pupil dilation response. In contrast, no significant effect was found in the ID group or the TD infant group, with ID showing a smaller but non-significant effect (OR = 9.01, SE = 1.03, *p* =.133) and TD infant showing no effect (OR = 0.83, SE = 0.32, *p* >.999). For analyses examining potential non-linearity in these effects, see Figure S1 in the Supplementary Material.

**Table 5:** Odds ratios for pupil response and the likelihood of not shifting attention

Fixed effects	OR (95% CI)	SE	*p*-value
*WS*			
Pupil response	**16.87 (3.86–73.56)**	**0.75**	**0.001**
*ID*			
Pupil response	9.01 (1.92–68.09)	0.03	0.133
*TD infant*			
Pupil response	0.52 (0.15–1.86)	0.65	> 0.999
*TD*			
Pupil response	**39.28 (4.33–355.66)**	**1.12**	**0.004**

*C* Confidence interval, *SE* Standard Error, *ID* Intellectual Disability, *TD* Typically Developed, *WS* Williams Syndrome. *P*-value is corrected using the Bonferroni method. Bold indicates a p-value below 0.05

## Discussion

Several studies indicate that individuals with WS have visual attention disengagement difficulties [6, 10, 12, 29] and an altered regulation of arousal [13, 16–18]. However, evidence for this claim comes primarily from studies using social stimuli, which may be particularly rewarding for individuals with WS, thus affecting their attention orienting [35]. Furthermore, the evidence for a specific challenge with disengagement as compared to shifting of attention is primarily indirect, as most studies did not use tasks that allow these to be separated. The present study reexamined disengagement and alerting in WS in one of the largest studies to date, using an experimental task designed to directly contrast disengagement and shifting. Our findings demonstrate that individuals with WS show an atypical pattern of visual attention that extends beyond social stimuli, characterized by slower gaze shifts and reduced likelihood of shifting attention than TD individuals. Children and adults with WS were also slower to shift their gaze than TD infants, suggesting that these alterations do not merely reflect a delay in the typical developmental trajectory. However, our results do not support the notion that these alterations are specific to WS. In fact, individuals with WS were *more likely* to shift than the ID group as a whole. Importantly, our results also contradict the hypothesis that WS is associated with a specific challenge with disengagement, and instead indicate that atypical attention in WS is primarily driven by challenges in *shifting* regardless of whether *disengagement* is needed. This aligns with the results shown by D’Souza et al. [26], who also found that toddlers with WS did not have specific difficulties with attention disengagement. We also found evidence of a link between arousal and visual attention in WS, which was distinct from the ID group of mixed genetic origin.

Contrary to our second hypothesis, a phasic alerting cue did not facilitate disengagement in WS. This argues against the notion that difficulties with disengagement in individuals with WS can be explained by low tonic arousal. Instead, the pupil dilation results suggest that arousal and visual attention are coupled in a relatively typical way in WS, similar to TD individuals, but not observed in the general ID group. On a group level, individuals with WS showed larger pupil responses to auditory warning signals preceding the visual stimuli than the ID group, but did not differ from age-matched TD individuals. These results indicate a more typical phasic arousal response to sensory stimuli in WS individuals.

The TD infant group was the only group to show a significant phasic alerting effect, as indicated by reduced gaze shift latencies in the overlap cued condition. Interestingly, aside from this distinction, the TD and TD infant groups exhibited broadly similar trends in gaze shift latency. Although no statistically significant differences were observed between these groups, this should not be interpreted as evidence of equivalence. Nonetheless, the comparable pattern of results may tentatively support the use of the TD-infant group as a reference point in studies of reflexive attention. Such trends could provide valuable insight into the development and underlying mechanisms of attention and how they might differ in clinical populations.

Additionally, we found that greater phasic pupil dilation following the alerting cue strongly predicted the likelihood of failing to shift attention in individuals with WS and TD children and adults, but not in the other groups. Interestingly, this behavioral pattern is consistent with findings of Keehn et al. [66] who observed a similar outcome, more no-shift trials, in autistic children with larger resting pupil size, a marker of high tonic arousal. They further suggested that altered arousal regulation could result in reduced or absent phasic LC-NE activation for peripheral targets, affecting sensory selection. While their study focused on tonic, baseline arousal, and ours on phasic arousal responses to an alerting cue, both point toward a common mechanism linking heightened arousal with impaired attentional shifting. For future research, it would be interesting to investigate the link between attention and sensory selection in WS further.

Both the WS and ID groups demonstrated difficulties shifting attention to the periphery even when disengagement was not required. In addition, the ID group showed a greater difficulty than the WS group and a reduced pupil response to the alerting cue. These attentional challenges were further reflected in slower gaze shift latencies in all conditions compared to the TD groups. The slower responses may indicate underlying arousal dysregulation, previously associated with less efficient attentional processes and altered adaptation of LC-NE activity, as seen in conditions like ADHD and autism [67]. Within the predictive coding framework [36], our findings may reflect a failure to generate accurate predictions of spatial attention, which would support efficient orienting. Since effective attention shifting is critical for learning, difficulties in this area result in fewer opportunities to engage with relevant information [21], thereby limiting learning outcomes. While our findings emphasize shifting efficiency, it is important to note that broader attentional control also includes the ability to resist distraction by irrelevant stimuli, which supports learning, especially in contexts with competing stimuli.

Decreased disengagement efficiency is a key aspect of the attentional profile in WS, especially in relation to faces [12], but our findings extend this to non-social stimuli as well. This supports the idea that attention regulation challenges in WS are not limited to socially salient cues, in line with Van Herwegen’s [9] hypothesis of domain-general disengagement difficulties. Similar patterns have been observed in other genetic syndromes associated with ID, such as Down syndrome [68] and Rett syndrome [57], suggesting a broader, underlying feature of attention regulation in neurodevelopmental conditions. These general attention difficulties may also contribute to the higher prevalence of autistic features and socio-communicative difficulties reported in WS [69–71], consistent with evidence linking disengagement inefficiency to core symptoms of autism [72]. However, while decreased disengagement efficiency is a prominent feature in these conditions, the way they impact social attention may vary. This broader pattern of disengagement efficiency could have important implications for social cognition and communication abilities, indicating that the difficulties with social attention in WS may not be isolated to face processing but instead reflect a more generalized issue with attention shifting.

Our results contribute to the understanding of the attention profile in WS, highlighting both unique and shared characteristics compared to other neurodevelopmental conditions such as ADHD and autism, which show phenotypically overlapping challenges. Since the benefit of an auditory phasic alerting effect has been evident in other neurodevelopmental conditions in childhood, it would be interesting to explore if this effect is present, and beneficial, in WS in an earlier developmental period. Studying attention in genetic conditions associated with attention difficulties can increase our understanding of the neural basis and the more basic functions of the attention networks in other symptom-based psychiatric diagnoses, such as ADHD.

### Limitations and directions for future studies

There are some limitations worth noting in this study. To begin with, the data was collected using two different eye-tracking systems. While the WS, ID, and TD groups were assessed using both systems (i.e., the systems were distributed across participants within these groups), the TD infant group was tested exclusively using a single eye tracker. Although data processing and calibration procedures were standardized across systems, we cannot entirely rule out the possibility that hardware differences may have introduced some variability. In addition, we did not have IQ data for all the participants with WS. Therefore, we are unable to comment on the potential impact of the developmental level on the phasic alerting cue for individuals with WS. However, in studies involving children with and without neurodevelopmental or genetic conditions, saccadic reaction times were not found to be associated with non-verbal intellectual ability [73], developmental quotient [74], or fine and gross motor skills [26]. The TD comparison groups with participants in different developmental stages help us acquire more understanding regarding the unique attention profile and phasic alerting in WS. Since this study concentrated on an ability that develops early in life (orienting attention), neither lack of IQ data nor the broad age range in the WS group should pose an issue. Although we lack IQ data for all participants, we have a comparable ID group regarding the level of global adaptive functioning. Furthermore, comparing to an infant group also yields insights into attention in WS. Additionally, exploring the relationship between ADHD symptom levels, autistic traits, and pupil dilation in individuals with WS would be an interesting focus for future studies. Keehn et al. [34] also proposed an intriguing hypothesis that the randomization of gap and overlap trials within the same block might be effortful for participants due to the need to switch between trial types and cope with unpredictability. This, in itself, could lead to slower saccadic reaction times and a higher proportion of no-shift trials. It is known from behavioural studies that cognitive flexibility and set-shifting are a challenge for individuals with WS [75, 76]. In line with the hypothesis by Keehn et al. [66] a higher number of no-shift trials and slower gaze shifts in the WS group are expected.

Our measures included stimuli with subtle movements in order to help maintain attention on the screen. Future studies should more systematically assess how different types of stimulus movement, such as blinking, flickering, or motion cues, may differentially influence pupil dilation, particularly in relation to low-level perceptual processing.

While our primary approach relies on between-group comparisons, future studies could more directly assess the link between attentional processes and individual differences in behavioral phenotype characteristics, such as ADHD symptoms or autistic traits, in WS through correlational or longitudinal designs. In this context, it would also be valuable to investigate the relationship between tonic and phasic pupil dynamics in genetic syndrome populations, as this may provide deeper insight into underlying arousal and attentional mechanisms.

## Conclusions

This study provides new insights into visual attention and arousal-based regulation in WS. While previous research has suggested that individuals with WS have difficulties with disengagement and arousal regulation, much of this evidence comes from studies using social stimuli. By directly contrasting disengagement and shifting of attention, our findings demonstrate that altered visual attention in WS extends beyond social stimuli and is primarily driven by difficulties with shifting regardless of whether disengagement is needed, alongside overall slower gaze shifts. Reduced attention shifting in WS may be partly explained by atypical arousal regulation. Additionally, our results indicate that attention difficulties in WS are not entirely syndrome-specific, as individuals with WS were more likely to shift than the ID group of mixed genetic origin. These findings contribute to a more nuanced understanding of attentional alterations in WS and their implications for social and cognitive development.

## Supplementary Information

Supplementary Material 1.

## Data Availability

The data that support the findings of this study are available from the corresponding author (AH) upon reasonable request following the ethics approval.

## Footnotes

1 A more complex model was conducted for the analyses on gaze shift latency and percentage no-shift trials but due to issues with convergence, and the simpler model generating the same results, the simpler model with only varying intercept for participant was chosen for those analyses. See Supplementary Material and Tables S2-S7 for the results of the different analyses and model comparisons.

2 For the TD group, the model had convergence issues due to little variation in the data. As can be seen in Table 3, mean percentage of no-shift trials were 5.59 (overlap silent) and 12.22 (overlap cued), meaning that this behavior was rare in this group.
