# Supplementary material for: Challenges with shifting, regardless of disengagement: attention mechanisms and eye movements in Williams syndrome
Source: J Neurodev Disord. 2025 Aug 13;17:48. doi: 10.1186/s11689-025-09639-z (PMC12344835; doi:10.1186/s11689-025-09639-z)
Supplement: Supplementary file 1 — Supplementary Material 1. [file 11689_2025_9639_MOESM1_ESM.docx]

**Supplementary Material**

**Results**

**Preliminary Analysis: Group Comparisons Mean Pupil Baseline**

Since normality was not met for pupil baseline (WS: *W* = 0.95, *p* <.001; ID: *W* = 0.97, *p* <.001; TD: *W* = 0.98, *p* <.001; TD-infant: *W* = 0.98, *p* <.001) Kruskal-Wallis test was conducted with Wilcoxon rank-sum test for follow-up pairwise comparisons. The Bonferroni method was used for multiple comparisons. There was a difference in mean pupil baseline across groups (χ^2^ (3) = 176.64, ***p* < .001). TD had a smaller mean pupil baseline than all groups (all groups *p* < .001) and WS had a smaller mean pupil baseline than both ID (*p* < .001) and TD-infant (*p* < .001). There was no difference between ID and TD-infant (p = .078).**

**Model Comparisons**

Fixed and random effects from the varying intercept for participant and varying slope for condition models for each outcome measure are presented in Table S1 (gaze shift latency), Table S3 (no-shift trials), and Table S5 (pupil dilation). Model fit comparison data are presented in Table S2 (gaze shift latency), Table S4 (no-shift trials), and Table S6 (pupil dilation). Model 1 refers to the varying intercept model and Model 2 refers to the varying intercept and varying slope model.

The more complex models with varying intercepts and slopes have singularity issues, both for gaze shift latency and no-shift trials. As can be seen in Table S1, the correlation between random slopes and intercepts for Participant OM and Participant OP is high (e.g., -0.79 and 0.97), and a similar pattern is observed in Table S4 (Participant OM -0.76 and Participant OP 0.95).

**Table S1**

*Fixed and random effects gaze shift latency presented in log(ms) and absolute value in ms. Intercept refers to group WS and condition Gap*

| **Fixed effect** | **Estimate (log(ms))** | **SE** | **df** | **z-value** | **p-value** | **Estimate back-transformed (ms)** |
| --- | --- | --- | --- | --- | --- | --- |
| Intercept | **5.90** | 0.05 | 122 | 131 | **<.001** | 364 |
| Condition OM | **0.19** | 0.03 | 116 | 5.74 | **<.001** | 438 |
| Condition OP | **0.01** | 0.03 | 110 | 6.34 | **<.001** | 445 |
| Group ID | 0.05 | 0.07 | 128 | 0.71 | .479 | 383 |
| Group TD | **-0.24** | 0.07 | 116 | -3.55 | **.001** | 285 |
| Group TD infant | **-0.26** | 0.07 | 127 | -3.68 | **<.001** | 281 |
| OM * ID | -0.08 | 0.06 | 127 | -1.37 | .172 | 462 |
| OP * ID | -0.03 | 0.06 | 122 | -0.71 | .482 | 469 |
| OM * TD | 0.03 | 0.05 | 101 | 0.61 | .544 | 343 |
| OP * TD | 0.06 | 0.05 | 95 | 1.33 | .186 | 349 |
| OM * TD infant | -0.06 | 0.05 | 131 | -1.13 | .259 | 339 |
| OP * TD infant | 0.02 | 0.05 | 124 | 0.40 | .692 | 344 |
| **Random effects** | **Variance** | **SD (log scale)** | **Corr.** |  |  |  |
| Participant Intercept | 0.07 | 0.27 |  |  |  |  |
| Participant OM | 0.02 | 0.14 | -0.79 |  |  |  |
| Participant OP | 0.02 | 0.14 | -0.63 | 0.97 |  |  |
| Residuals | 0.14 | 0.38 |  |  |  |  |

*Note*. OM = Overlap Silent, OP = Overlap Cued, ID = Intellectual disability, TD = Typically developed, SE = Standard error. Bold indicates a p-value below 0.05.

**Table S2**

*Model comparisons for the gaze shift latencies models*

| **Model** | **N parameters** | **AIC** | **BIC** | **logLik** | **Deviance** | **χ2** | **df** | **p-value** |
| --- | --- | --- | --- | --- | --- | --- | --- | --- |
| Model 1 | 14 | 4887.3 | 4978.3 | -2429.6 | 4859.3 |  |  |  |
| Model 2 | 19 | 4836.4 | 4959.9 | -2399.2 | 4798.4 | 60.91 | 5 | **<.001** |

*Note*. Model 1 refers to the varying intercept model. Model 2 refers to the varying intercept and varying slope model.

**Table S4**

*Fixed and random effects for percentage no-shift trials are presented in percentage units. The intercept refers to group WS and condition Gap*

| **Fixed effect** | **Estimate** | **SE** | **df** | **t-value** | **p-value** |
| --- | --- | --- | --- | --- | --- |
| Intercept | **20.22** | 3.38 | 121 | 5.99 | **<.001** |
| Condition OM | **4.91** | 1.93 | 1185 | 2.55 | **.011** |
| Condition OP | **11.26** | 2.03 | 95 | 5.54 | **<.001** |
| Group ID | 12.89 | 5.25 | 129 | 2.45 | **.016** |
| Group TD | **-16.97** | 5.21 | 119 | -3.25 | **.001** |
| Group TD infant | **-0.65** | 5.25 | 126 | -0.13 | .901 |
| OM * ID | 0.30 | 3.19 | 1411 | 0.09 | .925 |
| OP * ID | 34 | 3.33 | 115 | 0.10 | .919 |
| OM * TD | -2.55 | 2.91 | 1121 | -0.88 | .381 |
| OP * TD | -2.37 | 3.07 | 88 | -0.77 | .442 |
| OM * TD infant | 2.51 | 3.11 | 1409 | 0.81 | .420 |
| OP * TD infant | 1.58 | 3.25 | 113 | 0.49 | .627 |
| **Random effects** | **Variance** | **SD** | **Corr.** |  |  |
| Participant Intercept | 417.082 | 20.423 |  |  |  |
| Participant OM | 1.437 | 1.199 | -0.52 |  |  |
| Participant OP | 22.997 | 4.796 | -0.76 | 0.95 |  |
| Residual | 1277.429 | 35.741 |  |  |  |

*Note.* OM = Overlap Silent, OP = Overlap Cued, ID = Intellectual disability, TD = Typically developed, SE = Standard error. Bold indicates a p-value below 0.05.

**Table S5**

*Model comparisons for the percentage no-shift trials models*

| **Model** | **N parameters** | **AIC** | **BIC** | **logLik** | **Deviance** | **χ2** | **df** | **p-value** |
| --- | --- | --- | --- | --- | --- | --- | --- | --- |
| Model 1 | 14 | 63995 | 64090 | -31983 | 63967 |  |  |  |
| Model 2 | 19 | 63997 | 64125 | -31979 | 63959 | 8.09 | 5 | .151 |

*Note.* Model 1 refers to the varying intercept model. Model 2 refers to the varying intercept and varying slope model.

**Table S6**

*Fixed and random effects for pupil dilation are presented in mm. The intercept refers to group WS and the condition Overlap silent*

| **Fixed effect** | **Estimate** | **SE** | **df** | **t-value** | **p-value** |
| --- | --- | --- | --- | --- | --- |
| Intercept | **-0.026** | 0.006 | 3291 | -4.335 | **.001** |
| Condition OP | **0.061** | 0.010 | 91 | 5.849 | **<.001** |
| Group ID | 0.010 | 0.011 | 3291 | 0.923 | .356 |
| Group TD | 0.001 | 0.009 | 3291 | 0.068 | .946 |
| Group TD infant | 0.003 | 0.010 | 3291 | 0.262 | .793 |
| OP * ID | **-0.046** | 0.018 | 111 | -2.558 | **.012** |
| OP * TD | -0.003 | 0.016 | 84 | -0.172 | .864 |
| OP * TD infant | -0.014 | 0.016 | 105 | 0.840 | .403 |
| **Random effects** | **Variance** | **SD** | **Corr** |  |  |
| Participant Intercept | < 0.001 | <0.001 |  |  |  |
| Participant OP | 0.001 | 0.038 | NA |  |  |
| Residuals | 0.021 | 0.147 |  |  |  |

*Note.* OP = Overlap Cued, ID = Intellectual disability, TD = Typically developed, SE = Standard error. Bold indicates a p-value below 0.05.

**Table S7**

*Model comparisons for the pupil dilation models*

| **Model** | **N parameters** | **AIC** | **BIC** | **logLik** | **Deviance** | **χ2** | **df** | **p-value** |
| --- | --- | --- | --- | --- | --- | --- | --- | --- |
| Model 1 | 10 | -3383.4 | -3321.9 | 1701.7 | -3403.4 |  |  |  |
| Model 2 | 12 | -3369.2 | -3295.4 | 1696.6 | -3393.2 | 0 | 2 | >.999 |

*Note*. Model 1 refers to the varying intercept model. Model 2 refers to the varying intercept and varying slope model.

**Exploratory Pairwise Comparisons: Percentage of No-shift Trials**

In explorative within-group comparisons, all groups exhibited a significant difference with a higher percentage of no-shift trials in the overlap cued condition compared to the gap condition (all *p* < .001) and compared to the silent overlap condition (all *p* < .05). The WS and TD-infant groups had a significantly lower percentage of no-shift trials in the gap condition compared to the overlap silent condition (WS: *b* = -4,93, SE = 1.92, *p* = .031; TD-infant: *b* = -7,43, SE = 2.44, *p* = .006), however, this was not found in the ID and the TD groups (ID: *b* = -5.43, SE = 2.54, *p* = .097; TD: *b* = -2.38, SE = 2.17, *p* = .819).

In explorative pairwise between-group comparisons, in all experimental conditions, the TD group had a lower percentage unit of no-shift trials in comparison to all other groups (all *p* < .001). The WS group had a lower percentage unit of no-shift trials than the ID group in the overlap cued condition (*b* = -13.640, SE = 4.95, *p* = .039). In the gap and overlap silent condition, the difference in the percentage of no-shift trials was trending towards statistical significance (Gap: *b* = - 12.537, SE = 4.96, *p* = .074; Overlap silent: *b* = -13.036, SE = 4.96, *p* = .056) with the WS group having a lower percentage unit of no-shift trials than the ID group. There was no significant difference in percentage of no-shift trials in any condition for the WS and the TD-infant group (all *p* > .999), which was also the case for the ID and the TD-infant group (all *p* > .090). See Table S8 for pairwise comparisons.

**Table S8**

*Pairwise comparisons of percentage of no-shift trials in mean percentage units*

| **Group comparisons** | **Estimate** | **SE** | **df** | **t.ratio** | **p-value** |
| --- | --- | --- | --- | --- | --- |
| *Gap* |  |  |  |  |  |
| WS – ID | -12.54 | 4.96 | 186 | -2.53 | .074 |
| WS – TD | **16.92** | 4.91 | 168 | 3.45 | **.004** |
| WS – TD infant | 0.65 | 4.95 | 180 | 0.13 | > .999 |
| ID – TD | **29.46** | 5.33 | 180 | 5.53 | **< .001** |
| ID – TD infant | 13.19 | 5.37 | 191 | 2.46 | .090 |
| TD - TD infant | **-16.27** | 5.33 | 175 | -3.06 | **.012** |
| *Overlap Silent* |  |  |  |  |  |
| WS – ID | -13.04 | 4.96 | 187 | -2.63 | .056 |
| WS – TD | **19.48** | 4.93 | 171 | 3.95 | **< .001** |
| WS – TD infant | -1.85 | 4.93 | 178 | -0.38 | > .999 |
| ID – TD | **32.51** | 5.34 | 181 | 6.09 | **< .001** |
| ID – TD infant | 11.19 | 5.34 | 188 | 2.09 | .226 |
| TD – TD infant | **-21.33** | 5.31 | 174 | -4.01 | **< .001** |
| *Overlap Cued* |  |  |  |  |  |
| WS – ID | **-13.64** | 4.95 | 184 | -2.76 | **.039** |
| WS – TD | **19.48** | 4.90 | 167 | 3.97 | **< .001** |
| WS – TD infant | -0.82 | 4.90 | 173 | -0.17 | > .999 |
| ID – TD | 32.12 | 5.31 | 177 | 6.23 | **< .001** |
| ID – TD infant | 12.82 | 5.31 | 183 | 2.41 | .101 |
| TD – TD infant | **-20.28** | 5.27 | 168 | -3.85 | **.001** |

*Note.* SE = Standard error, TD = Typically developed, WS = Williams syndrome, ID = Intellectual disability. No-shift trials are presented in percentage units of trials where the participant does not shift their gaze to the periphery. Bold indicates a p-value below 0.05.

**Contrast of contrast comparison: Gaze Shift Latency**

Comparisons of within-condition differences across groups were conducted. No significant differences were found (all *p* > .702), indicating that the magnitude of the condition effects (e.g., gap vs. overlap silent) did not differ among any of the groups (Table S9).

**Table S9**

*Pairwise comparisons of within-group contrasts between groups (contrast of contrasts) in log(ms)*

| **Contrast** | **Estimate** | **SE** | **df** | **t.ratio** | **p-value** |
| --- | --- | --- | --- | --- | --- |
| *GM-OM* |  |  |  |  |  |
| WS-ID | -0.066 | 0.04 | 4846 | -1.61 | >.999 |
| WS-TD | 0.028 | 0.03 | 4813 | 0.85 | >.999 |
| WS-TD infant | -0.070 | 0.04 | 4823 | -1.83 | >.999 |
| ID-TD | 0.094 | 0.04 | 4839 | 2.29 | >.999 |
| ID-TD infant | -0.004 | 0.05 | 4842 | -0.09 | >.999 |
| TD-TD infant | -0.10 | 0.04 | 4815 | -2.56 | .702 |
| *GM-OP* |  |  |  |  |  |
| WS-ID | -0.033 | 0.04 | 4843 | -0.80 | >.999 |
| WS-TD | 0.061 | 0.03 | 4809 | 1.82 | >.999 |
| WS-TD infant | 0.011 | 0.04 | 4820 | 0.29 | >.999 |
| ID-TD | 0.094 | 0.04 | 4839 | 2.27 | >.999 |
| ID-TD infant | 0.044 | 0.05 | 4841 | 0.97 | >.999 |
| TD-TD infant | -0.050 | 0.04 | 4814 | -1.31 | >.999 |
| *OM-OP* |  |  |  |  |  |
| WS-ID | 0.032 | 0.04 | 4822 | 0.77 | >.999 |
| WS-TD | 0.033 | 0.03 | 4803 | 0.95 | >.999 |
| WS-TD infant | 0.080 | 0.04 | 4811 | 2.10 | >.999 |
| ID-TD | 0.000 | 0.04 | 4816 | 0.01 | >.999 |
| ID-TD infant | 0.048 | 0.05 | 4820 | 1.05 | >.999 |
| TD-TD infant | 0.048 | 0.04 | 4803 | 1.24 | >.999 |

*Note.* SE = Standard error, GM = Gap trials, OM = Overlap silent trials, OP = Overlap cued trials, WS = Williams syndrome, ID = Intellectual disability, TD = Typically developed. Estimates are presented in the difference of the difference in log(ms). Bold indicates a p-value below 0.05.

**Contrast of contrast comparison: Pupil dilation**

In the within-group comparisons, the ID group was the only group that did not demonstrate a greater pupil response in the overlap cued condition compared to the silent overlap trials. When comparing the contrast of contrasts across groups, it showed that the ID group exhibited a significantly smaller difference in pupil dilation between the overlap conditions compared to both the WS (Estimate = -0.044, SE = 0.02, *p* = .025) and TD-infant (Estimate = 0.057, SE = 0.02, *p* = .003) groups. Additionally, the ID group exhibited a marginally smaller difference when compared to the TD group (Estimate = 0.041, SE = 0.02, *p* = .056). No other between-group differences in contrast magnitude were found to be significant (all *p* > .999). See Table S10 for results.

**Table S10**

*Pairwise comparisons of within-group contrasts between groups (contrast of contrasts) on pupil dilation*

| **Contrast** | **Estimate** | **SE** | **df** | **t.ratio** | **p-value** |
| --- | --- | --- | --- | --- | --- |
| *OM-OP* |  |  |  |  |  |
| WS-ID | **-0.044** | 0.02 | 3425 | -2.87 | **.025** |
| WS-TD | -0.003 | 0.01 | 3373 | -0.23 | >.999 |
| WS-TD infant | 0.013 | 0.01 | 3384 | 0.96 | >.999 |
| ID-TD | 0.041 | 0.02 | 3417 | 2.60 | .056 |
| ID-TD infant | **0.057** | 0.02 | 3419 | 3.45 | **.003** |
| TD-TD infant | 0.016 | 0.01 | 3374 | 1.14 | >.999 |

*Note.* SE = Standard error, OM = Overlap silent trials, OP = Overlap cued trials, WS = Williams syndrome, ID = Intellectual disability, TD = Typically developed. Estimates are presented in the difference of the difference in mm. Bold indicates a p-value below 0.05.

**Can pupil dilation predict the likelihood of sticky fixation?**

We aimed to investigate whether statistical power and the potential for non-linear effects could influence the results. To explore this further, we visually examined smoothed plots of pupil response predicting the proportion of no-shift trials across groups (see Figure S1). These plots indicate possible non-linear relationships, particularly in the TD, TD infant, and ID groups. For example, the TD-infant group demonstrates a potential U-shaped pattern, while the TD group shows a steeper increase in no-shift probability at higher pupil responses. The ID group also exhibits a curvilinear trend, although it is more modest.

The fitted models show wide confidence intervals around the estimated effects. This is supported by large standard errors and wide confidence intervals for the odds ratios (WS: OR = 16.87, SE = 0.75, 95% CI = [3.87, 73.56], *p* = .001; ID: OR = 9.01, SE = 1.03, 95% CI = [1.19, 68.09], *p* = .133; TD: OR = 39.28, SE = 1.12, 95% CI = [4.34, 355.66], *p* = .004). While the linear effect in the TD group reaches statistical significance after Bonferroni correction, the very high odds ratio and wide confidence interval suggest uncertainty in the estimate. Similarly, the effect in the ID group is not statistically significant and exhibits wide uncertainty. The WS group also shows a large effect with a wide confidence interval, highlighting the need for caution in interpretation.

We additionally tested for non-linear effects by including a quadratic term for pupil response in each group’s model. In the TD infant group, the quadratic term was statistically significant (b = 6.09, SE = 2.67, *p* = .023), suggesting a potential U-shaped relationship, although the confidence intervals were still wide, reflecting limited certainty. In contrast, the quadratic term was not significant in any of the other groups (WS: b = 1.10, SE = 4.56, *p* = .810; ID: b = 5.41, SE = 6.17, *p* = .381; TD: b = 4.63, SE = 6.31, *p* = .464).

Taken together, while only the TD infant group showed a significant quadratic effect, the wide confidence intervals and large standard errors across the groups suggest that these non-linear patterns may be underpowered rather than absent. We therefore caution against interpreting the non-significant quadratic terms in the other groups as definitive evidence against non-linearity and acknowledge that more data may be needed to clarify the shape of the relationship.

**Figure S1**

*Pupil response predicting the proportion of no-shift trials across groups*


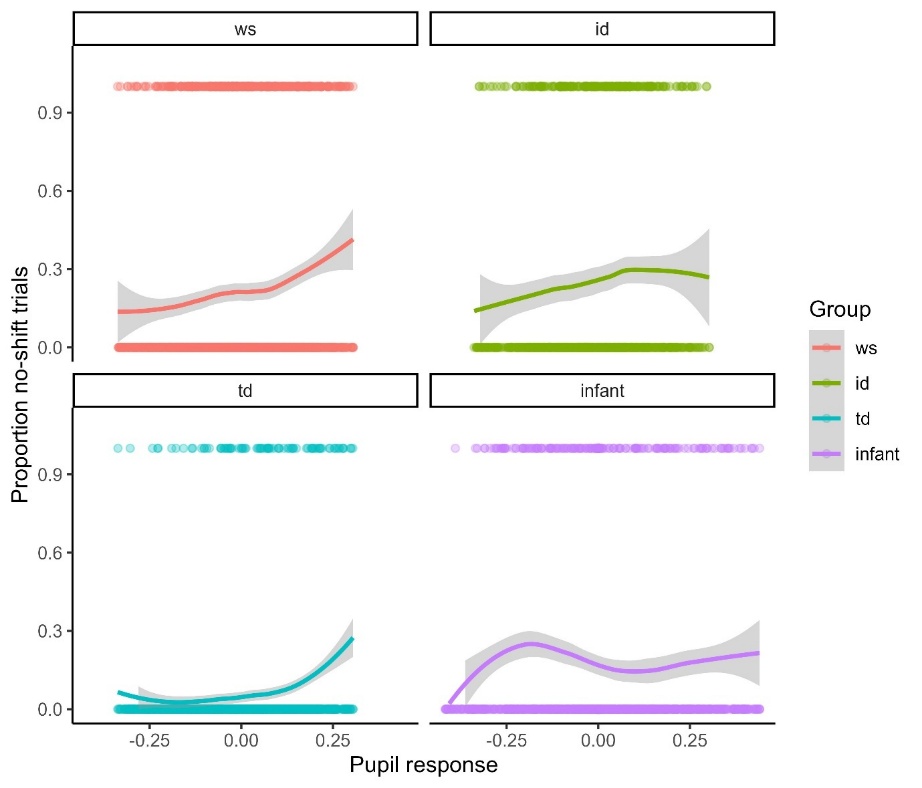


*Note.* WS = Williams syndrome, ID = Intellectual disability, TD = Typically developed. The shaded gray region surrounding the smoothed lines indicates the 95% confidence interval.
